# Factor XI inhibitors are the novel promising anticoagulants in the treatment of age related thrombotic disease

**DOI:** 10.3389/fcvm.2025.1498826

**Published:** 2025-05-30

**Authors:** Dandan Feng, Jianchun Wang

**Affiliations:** Department of Geriatrics, Shandong Provincial Hospital Affiliated to Shandong First Medical University, Jinan, Shandong, China

**Keywords:** thrombotic diseases, aged, factor XI inhibitors, anticoagulants, thrombosis, bleeding

## Abstract

The incidence and mortality of thrombotic diseases in the aged population are increasing year by year, which seriously affect the quality of life of the elderly. At present, antithrombotic drugs used in clinical practice have good efficacy, but they caused different degrees of age-dependent bleeding risk. Therefore, there is an urgent need to develop effective antithrombotic drugs with less risk of bleeding. Recent studies have shown that factor Ⅺ inhibitors can effectively reduce the incidence of thromboembolic events without increasing the risk of bleeding. Therefore, factor Ⅺ inhibitors are expected to be safe and effective new anticoagulants, providing a new sight for the prevention and treatment of thrombotic diseases. This paper reviews the biological functions of factor Ⅺ, the types and characteristics of factor Ⅺ inhibitors and the related research progress of factor Ⅺ inhibitors.

## 1 Introduction

Thrombotic diseases are caused by two pathological processes: thrombosis and thromboembolism, and are mainly manifested as venous thromboembolism (VTE), myocardial infarction, and ischemic stroke. Deaths caused by thrombotic diseases account for about 4% of global mortality, posing a great burden of public health (1). The elderly become the high-risk population of thrombotic diseases due to the changes of lifestyle and pathophysiological characteristics of the body. The main changes of physiological factors in the elderly are as follows: (1) the different degrees of endothelial damage and vascular sclerosis; (2) increased platelet aggregation; (3) increased blood viscosity; (4) hypercoagulation and increased coagulation factors (2). In addition, the risk factors of thromboembolism such as atrial fibrillation, malignancy, total knee arthroplasty, atherosclerosis, ischemic stroke, end-stage renal disease, and long-term bedridden are more common in the elderly. Therefore, The elderly are the key population in the prevention and treatment of thrombotic diseases.

Anticoagulant therapy is an important cornerstone for the prevention and treatment of thrombotic diseases. Traditional oral anticoagulants have commonly used in clinical practice, such as warfarin, which has good antithrombotic efficacy. But the dose-effect relationship of warfarin is affected by many factors. Close monitoring of international normalized ratio (INR) is necessary during warfarin treatment. Even if INR is maintained within the therapeutic range, there is still a high risk of intracranial hemorrhage (3–5). In recent years, the direct oral anticoagulants (DOACs) such as rivaroxaban, apixaban and dabigatran etexilate have better safety and efficacy than warfarin (6–9). However, bleeding was still considered an inevitable side effect of DOACs therapy, with the most common sites being the gastrointestinal and urinary tract (10–13). Due to the deterioration of physical functions, the elderly are not only with high risk of thrombosis, but also with high risk of bleeding during anticoagulant treatment. The high risk of bleeding limits the intensity of anticoagulant therapy tolerated by patients, leading to inadequate anticoagulation therapy, which increases the incidence of thromboembolic events. Therefore, it is of great clinical value to find a therapeutic target that effectively reduces thrombosis without increasing bleeding risk.

Epidemiological and numerous research data have shown that factor Ⅺ plays an important role in thrombosis, but little role in physiological hemostasis (14, 15). Therefore, factor Ⅺ has become an emerging and popular target for the development of new anticoagulants. The existing phase Ⅰ and phase Ⅱ clinical trial data indicated that compared with DOACs, factor Ⅺ inhibitors could be more effective to reduce the incidence of thromboembolic events without increasing the risk of bleeding (16). Therefore, factor Ⅺ inhibitors have significant potential value in the prevention and treatment of thrombotic diseases. In this review, we summarize the biological functions of factor Ⅺ, and the types and clinical research progress of factor Ⅺ inhibitors.

### 1.1 The biological functions of factor XI

**Figure 1:**
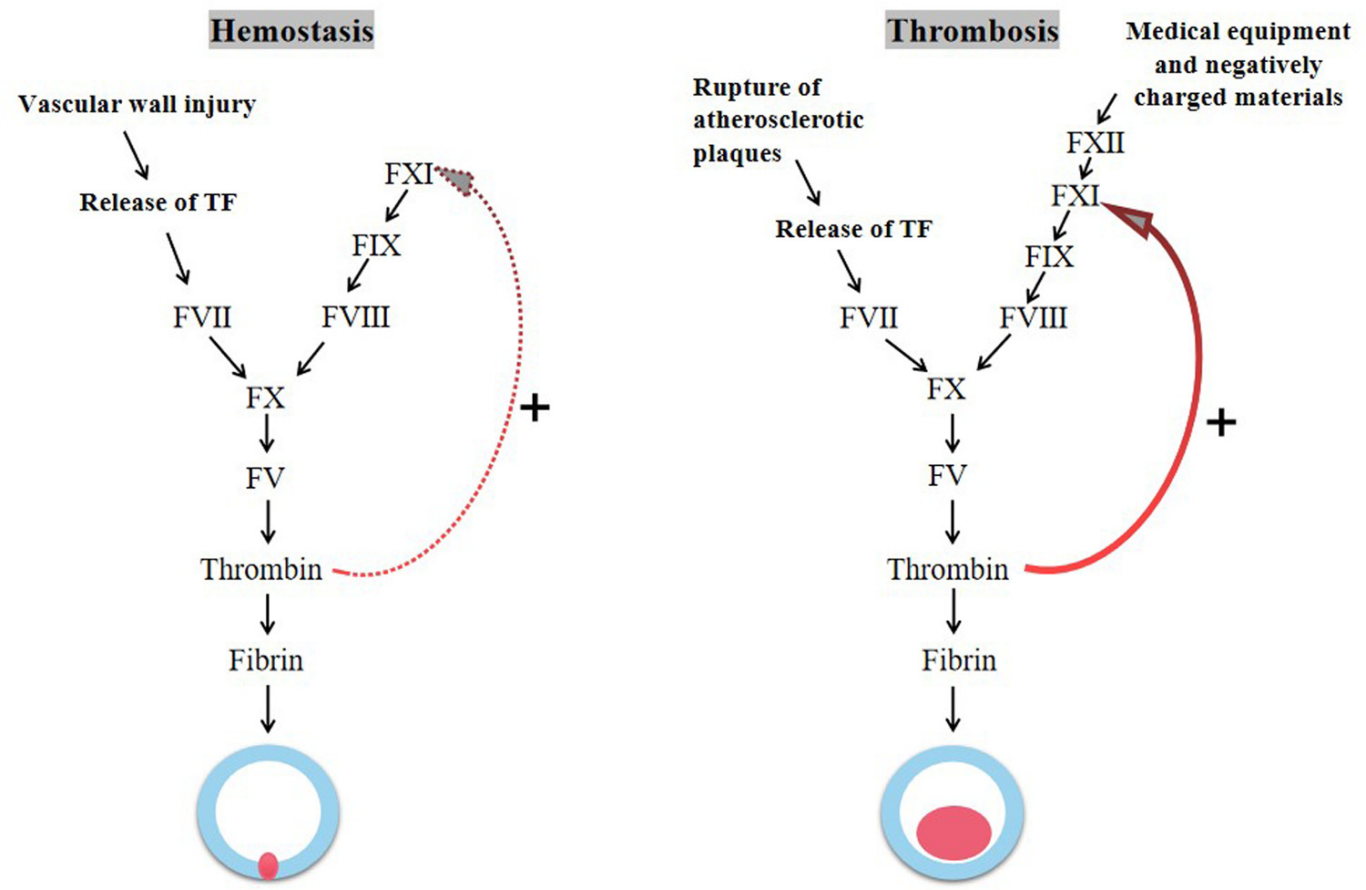
The process of hemostasis and thrombosis.

The same coagulation factors are involved in physiological hemostasis and thrombosis, but with two different outcomes (Figure 1). Hsu et al. suggested that factor Ⅺ may play a relatively limited role in hemostasis, and the activation of factor Ⅺ can reinforce hemostatic plug (16). Consistent with this view, most patients with hemophilia C (factor Ⅺ deficiency) do not have serious bleeding events, such as spontaneous bleeding, intracranial bleeding, intra-articular bleeding, and gastrointestinal bleeding. And bleeding events occurs only in certain individuals and under specific circumstances, for instance after surgery, tooth extraction, and urinary tract injury (17).

Different from hemostasis, the growth and propagation of pathological thrombosis appears to be largely dependent on the ability of factor Ⅺ to expand thrombin production. The activation of tissue factor (TF) following disruption of the vessel wall initiates coagulation causing a local burst of thrombin, which activates factor Ⅺ by positive feedback (18, 19). During the growth phase of thrombus, the naturally occurring negatively charged substance (e.g., DNA and polyphosphates) further activate factor Ⅺ via the contact pathway, amplifying thrombin formation (20–22). In addition, the activation of factor Ⅺ can inhibit tissue plasminogen activator (tPA)-induced blood clot fibrinolysis (23). At present, there are many epidemiological and preclinical research data supporting the key role of factor Ⅺ in thrombosis. Inhibition of factor Ⅺ can reduce thrombogenesis under pathological conditions in a variety of animal models (24–26). Patients with factor Ⅺ deficiency have significantly reduced risk of VTE and cardiovascular events (including myocardial infarction, stroke, and TIA) (17, 27, 28).

Because factor Ⅺ plays a crucial role in thrombosis and a minimal role in physiological hemostasis, targeted inhibition of factor Ⅺ has the potential to achieve the uncoupling of thrombosis and hemorrhage in anticoagulation therapy.

### 1.2 The types and characteristics of factor Ⅺ inhibitors

In the light of the pharmacological mechanism, factor Ⅺ inhibitors can be mainly divided into antisense oligonucleotides (ASOs), monoclonal antibodies, small molecule inhibitors (15, 23, 29). As shown in Table 1, both ASOs and monoclonal antibodies can only be administered parenterally, whereas small-molecule inhibitors can be administered orally. ASOs has a slow onset of action, requiring 3–4 weeks of administration to reduce factor Ⅺ to therapeutic levels. whereas monoclonal antibodies and small molecule inhibitors can take effect minutes or hours after administration. Monoclonal antibodies have a long half-life needing to be administered monthly once. Small molecule inhibitors have a short half-life, requiring administration once or twice daily. Moreover, ASOs and monoclonal antibodies are not metabolized by renal secretion and cytochrome P450 system, and are not substrates of P-glycoprotein, so they are ideal drugs for end-stage renal disease with a high risk of bleeding (30).

**Table 1:** Types and characteristics of factor Ⅺ inhibitors.

Types/Characteristics	ASOs	Monoclonal antibodies	Small-molecule inhibitors
Pharmacological mechanism	It binds to factor Ⅺ mRNA and blocks its protein expression	It binds to factor Ⅺ or factor Ⅺa and inhibits its biological function	It binds to factor Ⅺa and inhibits its biological activity
Method of administration	Subcutaneous injection	Intravenous or subcutaneous injection	Intravenous or oral administration
Frequency of administration	Weekly -Monthly	Monthly	Daily
Speed of onset	Slow (weeks)	Fast (hours–days)	Fast (minutes–hours)
Duration of effect	Weeks	Weeks	Minutes–hours
Renal excretion	No	No	No
Drug-drug interaction	No	No	Yes

### 1.3 Clinical research progress of factor Ⅺ inhibitors

#### 1.3.1 Factor Ⅺ-ASOs

ISIS 416858 (ISIS-factor ⅪRX) is an antisense nucleotide, which binds specifically to factor Ⅺ mRNA, inhibiting the expression of factor Ⅺ (31). Jeffrey et al. compared the efficacy and safety of different doses of ISIS-factor ⅪRX with enoxaparin in patients undergoing total knee arthroplasty (TKA). The results showed that, in terms of the prevention of VTE, the ISIS-factor ⅪRX 200 mg group was not inferior to enoxaparin group (27% vs. 30%), and the 300 mg group was significantly better than enoxaparin (4% vs. 30%). The rate of major or clinically relevant nonmajor bleeding was lower in the 300-mg dose group than that in the enoxaparin group (3% vs. 8%), but with no significant statistically difference. It is shown that 300 mg dose was more effective in reducing the incidence of TKA postoperative thromboembolism than enoxaparin, without increasing the risk of bleeding (32). In addition, an unfinished phase II clinical trial has been conducted to evaluate the proper dose of ISIS-factor ⅪRX and compare the safety and efficacy of the study drug to apixaban in end-stage renal disease (ESRD) patients requiring long-term hemodialysis (NCT03358030).

### 1.3.2 Monoclonal antibodies

#### 1.3.2.1 Abelacimab (MAA868)

Abelacimab is a fully human monoclonal antibody that binds to the catalytic domain of factor Ⅺ, thereby hindering its activation (33). Verhamme et al. published a study assessing the safety of different dose abelacimab vs. enoxaparin for the prevention of VTE in TKA patients. As for preventing VTE, 30 mg abelacimab was non-inferior to enoxaparin (13% vs. 22%), and the 75 mg and 150 mg abelacimab regimens were superior to enoxaparin (5%, 4% vs. 22%). The incidence of bleeding was 2%, 2%, 0%, and 0% in the 30 mg, 75 mg, 150 mg abelacimab, and enoxaparin groups, respectively. The results have shown that a single postoperative intravenous administration of abelacimab is more effective than enoxaparin in decreasing the incidence of VTE in TKA patients without increasing the risk of bleeding (34). A an ongoing phase II clinical trial of abelacimab (NCT04755283) that assess the efficacy and safety of abelacimab vs. rivaroxaban in patients with atrial fibrillation (AF) who have moderate-to-high risk of stroke.

#### 1.3.2.2 Osocimab (BAY 1213790)

Osocimab binds to a region near the active site of factor Ⅺa and inhibits its enzymatic activity (35). Weitz et al. evaluated the safety of various doses of osocimab compared with enoxaparin and apixaban for the prevention of VTE in TKA patients. The results showed that postoperative osocimab 0.6 mg/kg (15.7%), 1.2 mg/kg (16.5%) and 1.8 mg/kg (17.9%) were not inferior to enoxaparin (26.3%) for the incidence of VTE at 10–13 days postoperatively, whereas preoperative dose of 1.8 mg/kg of osocimab was superior to enoxaparin (11.3% vs. 26.3%). The incidence of bleeding for up to 10–13 days postoperatively was 0%, 1% and 3% in patients receiving postoperative 0.6 mg/kg, 1.2 mg/kg, 1.8 mg/kg osocimab, respectively, and 4.7% in patients receiving preoperative 1.8 mg/kg osocimab, 5.9% in those receiving enoxaparin. Hence, postoperative osocimab doses between 0.6 and 1.2 mg/kg appear to make the greatest clinical benefit (36). In addition, a phase II clinical trial is underway that evaluate the safety and tolerability of monthly subcutaneous administration of different doses of osocimab in ESRD patients undergoing regular hemodialysis (NCT04523220).

#### 1.3.2.3 Ab023 (Xisomab 3G3)

AB023 is a recombinant humanized antibody that binds to the apple 2 domain of factor Ⅺ and prevents its activation (37). A phase II study to evaluate the safety of a single administration of AB023 at the start of hemodialysis in patients with ESRD who need long-term hemodialysis with heparin intolerance. The results showed that the incidence of viral gastroenteritis had no difference between single-dose AB023 and placebo groups (12.5% vs. 12.5%), and AB023 treatment markedly decreased dialyzer clotting during heparin-free hemodialysis in ESRD patients (38).

### 1.3.3 Small molecule inhibitors

#### 1.3.3.1 Milvexian (JNJ70033093, BMS-986177)

Milvexian is a potent small molecule that binds to the active site of factor Ⅺa with high affinity and selectivity (39). Weitz et al. evaluated the efficacy and safety of milvexian compared with enoxaparin in patients with TKA. The results suggested that the incidence of VTE following twice-daily milvexian was significantly lower than enoxaparin (12% vs. 21%), and the dose response relationship with twice-daily milvexian was significant (21% taking 25 mg, 11% taking 50 mg, 9% taking 100 mg, and 8% taking 200 mg). The occurrence rate of bleeding had no difference between twice-daily milvexian and enoxaparin (4% vs. 4%). Studies indicated that oral milvexian was more effective than enoxaparin to prevent postoperative thromboembolism without increasing the risk of bleeding in TKA patients (40).

An uncompleted phase II trial to evaluate the safety and tolerability of a single oral dose of BMS-986177 in hemodialysis patients with ESRD (NCT03000673). And anthor phase II study (NCT03766581) is also underway to assess the efficacy of BMS-986177 for preventing new ischemic stroke or silent cerebral infarction in patients with acute ischemic stroke or transient ischemic attack (TIA).

#### 1.3.3.2 Asundexian (BAY 2433334)

Asundexian is a chemically synthesized and orally administered small molecule drug that can bind directly and strongly to the active site of factor Ⅺa, inhibiting its enzymatic activity. The phase I clinical trial conducted in healthy men showed that BAY 2433334 had good safety and tolerance, and dose-dependently inhibited factor Ⅺa activity and activated partial thromboplastin time (APTT) (41).

A phase II trial to determine the safety and the optimal dose of asundexian compared with apixaban in AF patients. Compared with apixaban, 20 mg and 50 mg asundexian significantly reduced resulted in lower rates of bleeding events in AF patients, with the near-complete inhibition of factor Ⅺa activity. This study provided a theoretical basis for phase Ⅲ clinical trial (42).

Shoamanesh et al. conducted a phase IIb trial to explore the efficacy and safety of asundexian in patients with acute noncardiac ischemic stroke. The results showed that the composite outcome of covert cerebral infarction and symptomatic ischaemic stroke occurred in 86 (19%), 99 (22%), and 90 (20%), 87 (19%) patients receiving asundexian 10 mg, 20 mg, 50 mg, and placebo, and the bleeding event was abserved in 19 (4%), 14 (3%), 19 (4%), and 11 (2%) patients in the asundexian 10 mg, 20 mg, 50 mg, and placebo group. Taken together, asundexian did not reduce the composite outcome of occult cerebral infarction or ischemic stroke and did not significantly increase the composite outcome of major or clinically relevant nonmajor bleeding. The efficacy outcome was not attenuated on account of no decrease in the incidence of covert cerebral infarction (75% of primary outcome events). Covert brain infarcts may be caused by underlying small-vessel disease that is no response to anticoagulation and is not affected by factor Ⅺ concentration. However, the results of *post hoc* analyses showed that treatment with asundexian 50 mg significantly reduced the composite outcome of recurrent ischemic stroke and transient ischemic attack in patients with co-existing atherosclerosis, providing theoretical basis for a phase III clinical trial (43).

A phase III clinical OCEANIC program was launched on August 28, 2022, namely OCEANIC AF and OCEANIC STROKE. It is expected to enroll up to 30,000 patients with AF and patients at high risk of non-cardiac ischemic stroke or transient ischemic attack in more than 40 countries to evaluate the efficacy and safety of asundexian for the prevention of stroke events (44).

## 2 Discussion

In the last decade, DOACs have been regarded as the optimal choice therpy for the prevention of thromboembolic events. It has been shown that DOACs were at least as effective as heparins and warfarin for preventing stroke and VTE, associated with lower rates of intracranial bleeding (45, 46). However, DOACs also have some limitations including: causing a high risk of bleeding, mainly gastrointestinal (46); areas where no clinical benefit was reported, such as the prevention of stroke in patients with mechanical heart valves (47), rheumatic heart disease-associated AF (48), transcatheter aortic valve implantation (49), the VTE prevention in patients with antiphospholipid antibody syndrome (50); no available data of DOACs in specific areas (e.g., kidney failure, thrombocytopenia, hypohepatia and extremes of body weight) (51, 52).

In recent years, factor Ⅺ has been an attractive target to explore the advantages of anticoagulation in specific clinical settings and overcome the limitations of the DOACs. Given the biological functions, targeting inhibition of factor Ⅺ can uncouple thrombosis from hemostasis and block the activation of the contact pathway in the process of thrombogenesis. The repression of factor Ⅺ seems to be rational in cases when thrombosis is triggered by artificial surfaces exposed to blood (e.g., haemodialysis circuits, mechanical valves, catheters, and cardiopulmonary bypass). At present, the research and development of factor Ⅺ inhibitors has been vigorously carried out, and a variety of factor Ⅺ inhibitors have entered the clinical trial stage (Table 2). A number of phase Ⅰ and phase Ⅱ clinical studies have indicated that factor Ⅺ inhibitors were more effective than DOACs for the prevention of VTE in patients with TKA and the improvement of dialyzer clotting in those with ESRD requiring long-term hemodialysis, without a significant increase in bleeding events (32, 34, 36, 38, 40). The safety of factor Ⅺ inhibitors compared with DOACs in AF patients has been researched (42), however, the differences in efficacy have not been studied. Among the factor Ⅺ inhibitors, abelacimab and asundexian have entered phase III clinical trails and have the potential to become novel safe and effective anticoagulants.

**Table 2:** Summary of clinical trials of factor Ⅺ inhibitors.

Type	Name	Disease type	Control group	Current state	Registration number	Journal of publication
ASOs	ISIS416858 (ISIS-FXI R_X_)	TKA	Enoxaparin	Phase Ⅱ clinical trial has been completed	NCT01713361	N Engl J Med
		ESRD requiring hemodialysis	Placebo	Phase Ⅱ clinical trial has been completed	NCT03358030	
Monoclonal antibodies	Abelacimab (MAA868)	TKA	Enoxaparin	Phase Ⅱ clinical trial has been completed	ANT-005 TKA, 2019-003756-37	N Engl J Med
		Atrial fibrillation	Rivaroxaban	Phase Ⅱ clinical trial has been initiated	NCT04755283	
	Osocimab (BAY 1213790)	TKA	Enoxaparin and apixaban	Phase Ⅱ clinical trial has been completed	NCT03276143	JAMA
		ESRD requiring hemodialysis	Placebo	Phase Ⅱ clinical trial has been completed	NCT04523220	
	AB023 (Xisomab 3G3)	ESRD requiring hemodialysis	Placebo	Phase Ⅱ clinical trial has been completed	NCT03612856	Blood
		Malignant tumors (requiring indwelling central venous catheter)	None	Phase Ⅱ clinical study is ongoing	NCT04465760	
Small-molecule inhibitors	Milvexian (JNJ70033093/BMS-986177)	TKA	Enoxaparin	Phase Ⅱ clinical trial has been completed	NCT03891524	N Engl J Med
		ESRD requiring hemodialysis	Enoxaparin and unfractionated heparin	Phase Ⅱ clinical trial has been completed	NCT03000673	
		Acute ischemic stroke or TIA	Placebo	Phase Ⅱ clinical trial has been completed	NCT03766581	
	Asundexian (BAY 2433334)	Acute noncardiac ischemic stroke	Placebo	Phase Ⅱb clinical trial has been completed	NCT04304508	Lancet
		Atrial fibrillation or noncardiac ischemic stroke	Apixaban or placebo	phase III clinical study has been initiated		

Moreover, the fact is that the average age of subjects was greater than 65 years in multiple studies involved in patients with TKA, AF, and acute noncardiac ischemic stroke (32, 34, 36, 40, 42, 43). Those studies have consistently shown that the efficacy of factor Ⅺ inhibitors was better than and without no increase in bleeding risk. Therefore, we can conclude that the factor Ⅺ inhibitors may be a promising anticoagulants in the treatment of thrombotic diseases in the elderly patients.

## 3 Challenges and future directions

Current factor Ⅺ inhibitors have varieties of advantages and limitations. Both ASOs and monoclonal antibodies have long half-lives, infrequent dosing, and stable efficacy, making them suitable for the prevention of cancer-associated thrombosis. However, these drugs may require the development of antidotes and reversal strategies. At present, there are potential difficulties in antagonising these drugs: the administration of factor Ⅺ concentrates can be thrombogenic, and prothrombin complex concentrates may not be sufficiently effective (53). ASOs take approximately 1 month to reach therapeutic levels and are therefore not suitable for acute situations. Among monoclonal antibodies, AB023 is an exception because of its short half-life and thus the potential for use in the periinterventional period, such as in the setting with indwelling catheter or extracorporeal circuit. ASOs and monoclonal antibodies are not metabolized by the kidney and therefore may be more suitable for patients with renal insufficiency. In addtion, the metabolism of small molecules depend on the liver, mainly interacted with cytochrome P450 3A4, thus, there may be potential drug-drug interactions (54).

Given the success of DOACs, factor XI inhibitors face a formidable challenge. there are some areas remaining to explore the safty and efficacy of factor XI inhibitors where DOACs have failed or where were not evaluated, such as stroke prevention in patients with mechanical prosthetic valves, rheumatic valve disease and TAVI, VTE treatment in patients with antiphospholipid antibody syndrome. Moreover, the effect of factor Ⅺ inhibitors in the setting of coronary thrombosis has not been elucidated (55). Ultimately, as with any new drug, the safety concerns may arise and have to be addressed. The long-term safety and effective antidotes of factor Ⅺ inhibitors need to be further investigated. In addition, the optimal dosage and safety profile in older populations remain to be established through ongoing and future large-scale phase III trials.
